# Applications of Probe Capture Enrichment Next Generation Sequencing for Whole Mitochondrial Genome and 426 Nuclear SNPs for Forensically Challenging Samples

**DOI:** 10.3390/genes9010049

**Published:** 2018-01-22

**Authors:** Shelly Y. Shih, Nikhil Bose, Anna Beatriz R. Gonçalves, Henry A. Erlich, Cassandra D. Calloway

**Affiliations:** 1 Children’s Hospital Oakland Research Institute, 5700 Martin Luther King Jr. Way, Oakland, CA 94609, USA; sshih@chori.org (S.Y.S.); nbose@ucdavis.edu (N.B.); herlich@chori.org (H.A.E.); 2 Forensic Science Graduate Program, University of California, Davis, 1909 Galileo Ct. Ste. B, Davis, CA 95618, USA; 3 Laboratório de Diagnósticos por DNA (LDD), Universidade do Estado do Rio de Janeiro (UERJ), Instituto de Biologia Roberto Alcantara Gomes (IBRAG), Rua São Francisco Xavier, number 524, Pavilhão Haroldo Lisboa da Cunha, 20550-900 Rio de Janeiro, RJ, Brazil; gonannab@gmail.com

**Keywords:** Next Generation Sequencing (NGS), Massively Parallel Sequencing (MPS), Probe Capture Target Enrichment, mitochondrial DNA (mtDNA), Single Nucleotide Polymorphism (SNP), Forensic Genetics, degraded DNA

## Abstract

The application of next generation sequencing (NGS) for the analysis of mitochondrial (mt) DNA, short tandem repeats (STRs), and single nucleotide polymorphism (SNPs) has demonstrated great promise for challenging forensic specimens, such as degraded, limited, and mixed samples. Target enrichment using probe capture rather than PCR amplification offers advantages for analysis of degraded DNA since two intact PCR primer sites in the template DNA molecule are not required. Furthermore, NGS software programs can help remove PCR duplicates to determine initial template copy numbers of a shotgun library. Moreover, the same shotgun library prepared from a limited DNA source can be enriched for mtDNA as well as nuclear markers by hybrid capture with the relevant probe panels. Here, we demonstrate the use of this strategy in the analysis of limited and mock degraded samples using our custom probe capture panels for massively parallel sequencing of the whole mtgenome and 426 SNP markers. We also applied the mtgenome capture panel in a mixed sample and analyzed using both phylogenetic and variant frequency based bioinformatics tools to resolve the minor and major contributors. Finally, the results obtained on individual telogen hairs demonstrate the potential of probe capture NGS analysis for both mtDNA and nuclear SNPs for challenging forensic specimens.

## 1. Introduction

Next generation sequencing (NGS) has shown great promise in the analysis of DNA from degraded, limited, and mixed samples often encountered in forensic cases [1,2,3]. NGS platforms produce millions of reads for sequencing multiple samples in a single sequencing run, thereby making it possible for sequencing of the whole mitochondrial (mt) genome and proving valuable for analyzing nuclear single nucleotide polymorphism (SNPs) and short tandem repeats (STRs) [4,5,6,7]. Current NGS technologies are equipped with sequencing chemistry capable of generating 25 million–3 billion reads per sequencing run with an output of ~15–600 Gb file size, providing sufficient sequencing depth for robust SNP and STR variant calling [6,8]. NGS analysis of STR polymorphism has revealed additional sequence polymorphisms within tandem repeats not identified by conventional capillary electrophoresis approaches [9,10]. Thus, NGS allows analysis of both length and sequence polymorphism on a single sequencing platform [11]. NGS applications have revolutionized not only STR and SNP typing, but also sequencing of small genomes such as the whole human mtgenome [12,13]. Analyzing the whole mtgenome, rather than just the hypervariable polymorphic regions HVI/HVII, would increase the discrimination power and alleviate the problem of common sequences found in the HVI/HVII regions shared by populations of the same origin [2]. In addition, NGS can increase resolution of mtgenome analysis by detecting low level-mixtures such as heteroplasmy (<5%), which would not be detectable using current standard analyses of mtDNA polymorphism by Sanger sequencing with its limit of detection at ~10–20% [13,14,15,16,17,18]. By utilizing this massively parallel sequencing aspect of NGS, the discrimination power can be increased with simultaneous analysis of multiple, various genetic markers including not just STRs, but also SNPs and mtDNA.

Sequencing SNPs and mtDNA can aid in genetic analysis of DNA from forensic samples when STR typing with capillary electrophoresis is not suitable, which is often the case for degraded DNA from forensic samples due to biological, environmental, or oxidative degradation. In these cases, the DNA is highly fragmented, and therefore both primer binding sites may not be present in most template DNA molecules, rendering the amplification of SNPs or STRs impractical [11,19] The inability to amplify large amplicons would result in partial genetic profiles with dropout of alleles or loci [19,20]. Furthermore, mixtures, particularly of trace DNA, may represent a significant challenge for genetic analysis due to stochastic variation and potential allelic dropout [2,21]. Alternative approaches such as miniSTR analysis can be used for degraded DNA samples by designing primer binding sites that are closer to the target regions, thereby reducing the amplicon size to 80–150 bp [20,22,23]. Another useful approach is by typing SNPs with primers immediately adjacent to targeted single base variations, thereby reducing the amplicon size to as short as ~50 bp [11,24]. The short SNP amplicon sizes are particularly useful for analysis of DNA from degraded forensic specimens, such as those in mass disaster cases, and clinical samples, such as fetal cell free DNA (cfDNA) in maternal blood [11,25,26]. In addition to SNPs, the analysis of mitochondrial sequence polymorphism has proved useful when nuclear DNA is too degraded since mtDNA is more likely to be present due to its high copy number in forensic biological samples, such as telogen hairs roots, hair shafts, bones, teeth, and touch DNA [14,27]. Mitochondrial DNA, as a haploid lineage marker, is also valuable for the de-convolution of mixtures since each individual contributes only one sequence rather than two alleles. Presence of two nucleotides in a single mtgenome position would either indicate a mixture or presence of heteroplasmy [14,28]. The non-recombining uniparental inheritance of mtDNA is also useful for analyzing samples from mass disasters or missing person cases, in which a missing individual’s mitochondrial haplotype can be directly compared to the haplotype of the individual’s maternal relatives [13,14,27].

However, designing primers for mtgenome sequencing can be challenging for these densely polymorphic regions [29]. Furthermore, multiplexing SNPs using PCR based approaches still requires presence of intact primer binding sites. Therefore, enrichment of target DNA for forensically challenging samples by using an alternative method that does not depend on the presence of intact primer binding site would improve recovery of fragmented DNA prior to deep sequencing [4,5,6]. One alternative strategy is target enrichment using synthetic probes [30]. In general, the probe capture enrichment process consists of hybridizing biotinylated DNA or RNA probes to complementary DNA fragments in a shotgun library either in solution or on a solid surface [7]. The target DNA fragments are bound to the probes while the unbound DNA, primer dimer artifacts, and impurities are washed away [30,31,32]. These capture technologies using synthetic probes have been widely applied for exome capturing and sequencing in research and clinical laboratories [4,5,33,34].

Currently, several capture enrichment technologies are commercially available, including Agilent’s capture platforms, Roche NimbleGen SeqCap EZ platforms, and Illumina TruSeq DNA and Nextera Exome platforms [34,35]. Several groups have conducted studies comparing some of these capture enrichment platforms for their probe type, probe length, probe design, and target enrichment efficiency [34,36]. Agilent technology uses RNA probes with probe length ~120 bp while Illumina and NimbleGen technologies employ DNA probes that are, respectively, ~95 bp and ~55–105 bp in length [35,36]. The probe design for Agilent uses probes that are adjacent to each other to cover the region of interest. Illumina’s design uses paired end reads that extend to cover the region of interest. NimbleGen’s design uses a high density of probes that overlap target regions, resulting in a high redundancy of probes per base [34,35]. Clark et al. demonstrated that target enrichment efficiency was the highest for NimbleGen at 96.8% at ≥10× read depth, followed by Illumina at 90.0% at ≥10× read depth and Agilent at 89.6% at ≥10× read depth [35]. Sulonen et al. also showed that NimbleGen had the highest target efficiency of the two capture platforms compared (NimbleGen and Agilent) [36]. Based on probe design and performance differences, one may choose one target enrichment platform over the other. Our evaluation of these results led to the decision of choosing Roche NimbleGen SeqCap EZ platform for our custom probe capture panels owing to its tiling approach for probe design and higher enrichment efficiency. With high redundancy of probes per base, this design can efficiently and uniformly target regions with high degree of polymorphism, such as the mtgenome regions and nuclear SNPs [29].

Hybrid probe capture NGS assays have proved useful for capturing and sequencing ancient DNA (aDNA) from human remains [37,38]. The DNA from ancient remains is often highly degraded due to chemical or environmental damages and can be highly contaminated with exogenous DNA from the microbes and organisms that colonize the remains in the burial environment. Exogenous DNA introduced in laboratories or by personnel during various stages of storage, cleaning, extraction, and library preparation can also be a source of contamination [39]. Thus, endogenous DNA from these ancient samples can comprise as low as ~1–5% of the total DNA, such as the cases with the Neanderthal DNA samples. Using the 454 pyro-sequencing platform, Green et al. successfully mapped the complete Neanderthal mtgenome from 70 Neanderthal bone samples [40,41,42]. However, to capture all target DNA fragments from these shotgun libraries without enrichment would require too many sequencing runs [39,43]. This limitation can be overcome by applying a probe capture enrichment method as demonstrated in the study by Reich et al. whereby using a target enrichment method resulted in recovery of ~70% endogenous DNA from an ancient Denisova phalanx sample dated ~50,000–~30,000 years ago [44].

Similar to ancient DNA, DNA from forensic, mass disaster, or missing person case samples is often limited, highly degraded, and mixed. Recently, hybrid probe capture NGS technologies successfully recovered mtgenome DNA fragments from forensically challenging samples such as modern bones and hair shafts [1,2]. Templeton et al. showed that in-solution hybridization with DNA probes generated from human mtDNA yielded full coverage of the mtgenome from ~2500 years old bone samples with short mtDNA fragments (77 bp) and low mtDNA copies (350 copies/µL) [2]. Eduardoff et al. also demonstrated the efficiency, robustness, and sensitivity of the probe capture method on recovery of short mtDNA fragments (50 bp) from hair shafts and ancient solid tissues [1]. The primer extension capture (PEC) NGS method used by Eduardoff et al. was originally designed to target the mtDNA control region (CR). Their results demonstrated coverage of partial and full mtgenome, with noticeably lower coverage per base in regions outside of the CR, as expected [1]. In addition to biotinylated DNA probes, RNA probes can also be used for enriching limited and fragmented DNA from ancient and forensic specimens [33,39].

Here, we present the results obtained with our custom probe capture panels for target enrichment of the whole mtgenome and 426 nuclear SNPs in conjunction with deep sequencing on the Illumina MiSeq platform from a single shotgun library demonstrating the application to the analysis of limited, degraded, and mixed forensic samples [45,46]. Both custom probe capture panels were designed using the Roche NimbleGenSeqCap EZ custom probe capture assay. With its redundant, overlapping tiling design, the DNA probes can efficiently hybridize and capture highly fragmented DNA and densely polymorphic regions such as the HVI/HVII regions for clonal, massively parallel sequencing [34]. This redundancy of sample tiling using DNA probes greatly improves the capture of forensically challenging DNA samples such as DNA from telogen hair roots, telogen hair shafts, and touch-DNA. This strategy allows the capture of both mtDNA and nuclear SNP markers from a single shotgun DNA library without consuming additional DNA extracts of limited forensic biological samples.

## 2. Materials and Methods

### 2.1. Sample Preparation

Samples were prepared for the following studies as described below: mtDNA sensitivity study, nuclear DNA sensitivity and size selection, mock degradation, mtDNA mixture, and telogen hairs study.

#### 2.1.1. Mitochondrial DNA Sensitivity Study

**Figure 1:**
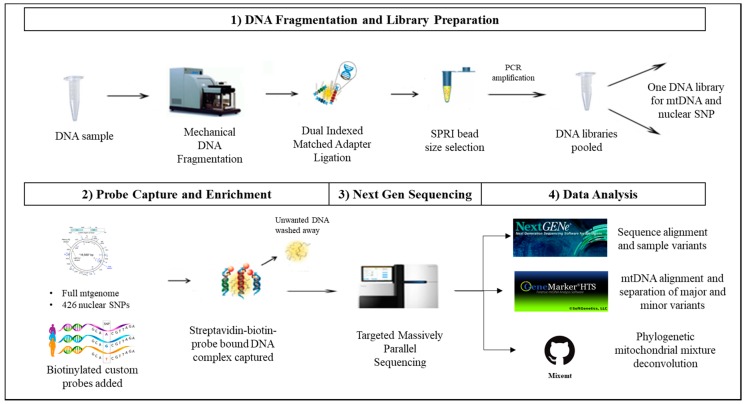
Novel probe capture next generation sequencing (NGS) assay for sequencing mitochondrial (mt) and nuclear DNA. To circumvent choosing between mitochondrial and nuclear analysis of degraded or limited forensic DNA samples, a capture enrichment based library preparation method can be implemented. In this method: (**1**) DNA libraries are prepared by fragmenting the DNA samples using Covaris^®^ M220 Focused-ultrasonicator^TM^ (Covaris). DNA fragments are ligated with dual-index adapters, and the amplified DNA libraries are size-selected using SPRIselect^®^ beads (Beckman Coulter Life Sciences, Indianapolis, IN, USA). (**2**) Shotgun libraries are enriched using a DNA probe based method of targeted capture enrichment for the whole mtgenome and eight categories of nuclear single nucleotide polymorphism SNP markers (426 SNPs). (**3**) The enriched samples are then sequenced on a NGS platform. (**4**) The nuclear SNP data is analyzed using NextGENe (SoftGenetics LLC, State College, PA, USA), while the mitochondrial data is analyzed using NextGENe, GeneMarker^®^HTS (SoftGenetics LLC), and Mixemt [50].

Dilutions targeting input DNA amounts of 1 ng, 100 pg, and 10 pg were prepared using the control human HL-60 DNA SRM2392-I (NIST, Gaithersburg, MD, USA). These samples were processed following our standard DNA fragmentation and library preparation as described below and depicted in Figure 1.

#### 2.1.2. Nuclear DNA Sensitivity and Size Selection Study

The single source male control DNA NA24129 (Coriell Institute for Medical Research, Camden, NJ, USA) was mechanically fragmented using the Covaris^®^ M220 Focused-ultrasonicator^TM^ (Covaris, Woburn, MA, USA) to an average size of 175 bp with a range from 25 bp to 250 bp. To test short template sample analysis, size selection was carried out at ≤75 bp using the Pippin Prep^®^ (Sage Science, Beverly, MA, USA). DNA libraries were then prepared for the ≤75 bp size selected fragments with 10 ng, 1 ng, and 0.5 ng DNA sample input amounts. The detailed procedure for the preparation of these size selected samples is described in the study by Bose et al. [46].

#### 2.1.3. Mock Degradation Study

For this study, 1 ng of control DNA (K562 for mtDNA and 2800M for nuclear SNPs (Promega, Madison, WI, USA) was mechanically fragmented to 150 bp to simulate degraded samples following the manufacturer’s protocol (Part No. 010166 Rev E, Covaris). These artificially degraded samples were then processed following our standard DNA fragmentation to 250 bp and library preparation as described below. 

#### 2.1.4. Mitochondrial DNA Mixture Study

For the mtDNA mixture, a two-person mixture was prepared in vitro using DNA from two previously extracted blood samples (Caucasian C163 and US Hispanic H104) of known mitochondrial haplotypes targeting a total input of ~300,000 mtDNA copies. The nuclear DNA and mtDNA amounts were quantified using a duplex real-time quantitative PCR (qPCR) assay [47,48]. The mixture samples were processed following our standard library preparation procedure as described below.

#### 2.1.5. Telogen Hair Study

Two telogen hairs (shed hairs) were collected from female participants for the mock forensic sample study with the approval from the Institutional Review Board Administration (IRB # 852842) at the University of California, Davis, USA. The hairs were collected by combing and were screened for telogen roots using a transmitted light microscope. Two centimeters of the hairs were cut from the proximal end and labeled P1FTR2 and A1FTR1. The telogen hair roots were cleaned with 2% sodium dodecyl sulfate (SDS) followed by 10 min of sonication and a rinse of molecular grade water. The hair roots were then stained with Harris Hematoxylin (HE) (Richard-Allan Scientific 72704, Kalamazoo, MI, USA) and counted for nuclei under a transmitted light microscope [49]. For telogen hair P1FTR2, >60 nuclei were observed and for hair A1FTR1, 20–60 nuclei were counted. DNA from these telogen hair roots was extracted using the QIAamp DNA Micro Kit (QIAgen^®^, Valencia, CA, USA) following the manufacturer’s protocol with elution in 50 µL of TE-4 (pH 8.0, 0.1 M EDTA). DNA extracts were quantified using a real-time Taqman™ Degradation qPCR assay to detect both human nuclear DNA and mtDNA by targeting two nuclear DNA makers, nuTH01 (~170–190 bp), nuCSF (67 bp), and a 283 bp mtDNA marker [47,48]. The mtDNA marker approximates the number of mtDNA copies in DNA extracts. DNA libraries were prepared using 1 ng DNA as estimated by the nuTH01 marker with ~270,000 and ~540,000 mtDNA copies for P1FTR2 and A1FTR1, respectively.

### 2.2. DNA Fragmentation and Library Preparation

All DNA samples, except the samples in the nuclear DNA sensitivity and size selection study, were mechanically sheared to 250 bp following the manufacturer’s guideline for the Covaris^®^ M220 Focused-ultrasonicator^TM^ for the 50 µL screw capped tube. DNA libraries were then prepared using KAPA Hyper Prep Kit for Illumina^®^ platforms (KAPA Biosystems, Roche^®^, San Francisco, CA, USA) following the manufacturer’s protocol with optimized PCR cycle numbers (Table 1). Other modifications include two post-adapter-ligation SPRI^®^ cleanups at 0.8× bead-to-sample volume ratio and two post-amplification SPRI^®^ cleanups at 1× bead-to-sample volume ratio with SPRIselect^®^ reagent (Beckman Coulter Life Sciences, Indianapolis, IN, USA) [51]. For all samples, Matched Dual Index Adapters (Integrated DNA Technologies, Coralville, IA, USA) were used in DNA library preparations. The size of the amplified DNA libraries was determined using the Agilent 2100 Bioanalyzer and Agilent 7500 DNA kit (Agilent Technologies) to ensure that all amplified products were within the optimal size range (300–700 bp) for sequencing.

### 2.3. Probe Capture Enrichment and Next Generation Sequencing

Tecan^®^ Microplate Reader Infinite^®^ F200 instrument (Tecan, Männedorf, Switzerland) and Quant-iT PicoGreen^®^ dsDNA Assay Kit (Thermo Fisher) were used to quantify the DNA sample library amount for subsequent pooling. For whole mtgenome capture, up to 24 sample libraries with unique adapter index sequences, were pooled at equal DNA amounts for a total of 1 µg of DNA. For nuclear SNP capture, a total of 1 µg was targeted by pooling equal DNA amounts of 9–11 samples depending on sequencing read depth requirements per sample.

The mtDNA custom probe capture panel was developed by Calloway et al. using proprietary software by NimbleGen targeting the mtDNA majority consensus sequence from a global mtDB-Human Mitochondrial Genome population Database [29]. The probe design uses unique probes with a tiling approach, resulting in high redundancy of probes overlapping target sequences. Custom parameters were used and the final design selected directly targets most of the mtgenome with only two small gaps at positions 2506–2513 (7 bp) and 2962–2972 (10 bp). The DNA probes are 50–100 bp in size with an average of 35 different probes per base. The design and hybridization conditions allow the probes to tolerate up to five mismatches within a DNA probe [29]. The nuclear SNP probe capture panel developed by Bose et al. consists of 426 SNPs with high target capture efficiency, including 41 ancestry, 135 identity, 73 microhaplotypes, 22 phenotypically informative, 24 tetra- and 28 tri-allelic, and 24 X and 79 Y SNPs (Table S1) [46]. The probes are 50–100 bp in size and span over SNP coordinates with ±50 bp. The SNP coordinates were determined for human genome version GRCh37/hg19 using the UCSC Genome Browser [46]. 

The probe capture experiments were carried out using the Roche NimbleGen SeqCap EZ Library Developer Library Kit following the manufacturer’s protocol for both mtDNA and nuclear SNP capture (NimbleGen SeqCap EZ Library SR User’s Guide V.5.1) (Roche). The hybridization samples were prepared by combining COT human DNA (Roche^®^), hybridization enhancing oligo pool, and the previously pooled multiplex DNA libraries. The COT human DNA fraction is largely highly repetitive DNA elements used to increase the specificity of hybrid probe capture. The hybridization samples were then concentrated using a Jouan RC 10.22 Vacuum Concentrator Centrifugal Evaporator following the manufacturer’s protocol (Jouan, Inc., Winchester, VA, USA). The concentrated multiplex DNA libraries were hybridized to the custom probes overnight for 16–20 h. The hybridized products were enriched and amplified using KAPA HiFi HotStart Ready Mix (KAPA Biosystems) and Dynabeads™ M-270 Streptavidin (Thermo Fisher) following the manufacturer’s protocol (NimbleGen, Roche). The mtDNA hybridization products were amplified at 13 PCR cycles, and the nuclear SNP hybridization products were amplified at 15 PCR cycles.

The amplified enriched DNA library pool was quantified using both Agilent 2100 Bioanalyzer and Tecan^®^ Microplate Reader Infinite^®^ F200 instrument. The quantified enriched DNA library pool was then prepared using the “Preparing Libraries for sequencing on the MiSeq” protocol with a final concentration of 11 pM of DNA and 5–10% control phiX DNA. Sequencing on the Illumina MiSeq instrument was carried out following the manufacturer’s protocol (Illumina). The Illumina MiSeq Reagent v2 kit (500 cycles, 2 × 250 reads) was used for mtDNA sensitivity study, mock degradation study, mixture study, and forensic type sample study. The Illumina MiSeq Reagent v2 Kit (300 cycles, 2 × 150 reads) was used for the size selection study.

### 2.4. Data Analysis

Samples in the mtDNA sensitivity study were analyzed with and without the PCR duplicates using both GeneMarker^®^HTS v.1.2.2 and NextGENe^®^ v.2.4.2 (SoftGenetics LLC, State College, PA, USA). Nuclear SNPs were analyzed in the size selection and mock degradation studies using the NextGENe^®^ software. Telogen hairs were analyzed using GeneMarker^®^HTS for the whole mtgenome and NextGENe^®^ for SNP analysis.

Initial mtgenome sequencing analysis was carried out using the GeneMarker^®^HTS software by aligning the forward and reverse sequence reads (FASTQ) of each sample to the revised Cambridge Reference Sequence (rCRS) [52]. The software then outputs all forward and reverse reads (unmerged) into a single BAM file. Default motif was applied to help align data in problematic regions such as indels. Default identity setting was at 90% to prevent alignment of sequence reads that were <90% identical to the rCRS. Soft clipping at the 3’ end was at Q ≤ 25 to allow trimming of bases with quality scores below this threshold at the end of the reads. Additional custom filter settings were used: the variant percentage filter was set to ≥10% to filter out variants that did not meet the 10% threshold. Variant allele coverage filter was set at ≥10 so that each variant detected exhibited at least tenfold coverage. Total coverage was set at ≥100 (with the duplicates) to ensure that there is at least 100× coverage per base. In addition to the variant filters, allele score difference was set to ≤2.5 and the allele balance ratio was set to ≤5 for both SNP and Indel. Total reads, alignment percentage, average coverage, and the mtgenome coverage were obtained from the alignment statistics reports generated using GeneMarker^®^HTS. The PCR duplicate removal tool in GeneMarker^®^HTS removes PCR duplicates that have the same starting positions in the forward and reverse reads (reads not merged) [53].

Mitochondrial genome analysis using NextGENe^®^ v.2.4.2 was carried out by first converting FASTQ files to FASTA files. Default alignment (default homology at 85%) was used for samples with PCR duplicates; custom alignment with homology at 90 was used for samples without PCR duplicates. Custom filter settings were applied to all samples: 10% mutation percentage, three SNP allele count, and 100× total coverage. Depth of coverage and sequence read length distribution (average read length) were both obtained from reports generated using NextGENe^®^ v.2.4.2.

In addition to GeneMarker^®^HTS, the sequenced reads for the mtDNA mixture samples were analyzed using Mixemt developed by Vohr et al. [54]. Prior to analysis using Mixemt, the raw sequence reads were trimmed for adapters, and the overlapping reads were merged using SeqPrep [49]. Both merged and unmerged sequence reads were then aligned separately to the Reconstructed Sapiens Reference Sequence (RSRS) using the Burrows–Wheeler Aligner (bwa) tool, which also converts the FASTQ files to SAM files [51,52]. SAMtools was then used to collapse the PCR duplicates and convert SAM files to BAM files [55]. The BAM files were used for Mixemt analysis, which assigns each sequence read to a haplogroup based on the probability of the read originating from a contributing haplogroup [50,54].

For nuclear SNPs analysis, raw sequence data of all samples were analyzed using the SoftGenetics^®^ NextGENe^®^ Software v2.4.1.1 (SoftGenetics LLC). The raw sequence data was first converted from FASTQ files to FASTA files. The FASTA files of each sample were then aligned to version GRCh37/hg19 of the human genome in NextGENe. Default alignment settings were used. Coverage and allele percent of targeted SNPs was generated in the NextGENe Mutation Report tool by specifying SNP genomic locations (as a BED file). Further sequencing statistics such as diploid read depth per SNP and percent SNP coverage were derived from the mutation reports [46]. SNP coverage percent was established based on alleles exhibiting >10× reads and SNP loci exhibiting ≥20× reads, which is within the threshold necessary for confident SNP variation calling [4]. Locus and allele dropouts were determined for size selected samples based on comparison of the sequencing data from experimental samples to the sequencing data from 25 ng references.

## 3. Results

### 3.1. Mitochondrial DNA Sensitivity Study

**Figure 2:**
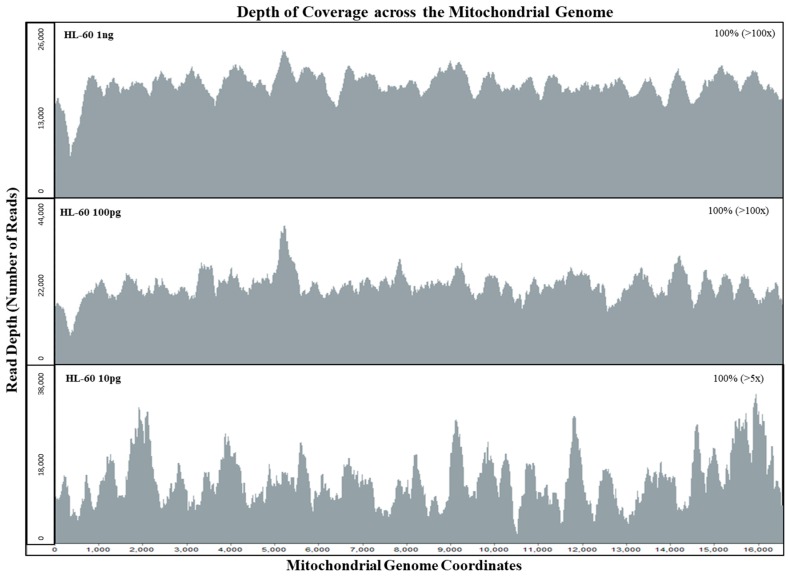
Depth of coverage across the whole mitochondrial genome for mtDNA sensitivity study samples. Limiting dilution of HL60 control DNA samples were tested with input DNA amounts of 1 ng, 100 pg, and 10 pg for the mtDNA sensitivity study. All HL60 samples exhibited full coverage of the mtgenome at >5× read depth (PCR duplicates removed using NextGENe^®^ v.2.4.2).

The sensitivity of the mtgenome probe capture assay was tested with HL60 samples at DNA amounts of 1 ng, 100 pg, and 10 pg. Sequencing data of the whole mtgenome was analyzed using GeneMarker^®^HTS with PCR duplicates included and removed. When PCR duplicates were included, all samples exhibited full coverage of the whole mtgenome at >100× read depth (Table 2). Even after removing PCR duplicates, both 1 ng and 100 pg samples exhibited full mtgenome coverage at >100× read depth (Figure 2). The 10 pg sample exhibited full coverage of the mtgenome at >5× read depth per base with an average coverage of 22× without PCR duplicates (Figure 2, Table 2). These results also demonstrated the specificity of our custom probe capture NGS method; of the average total of ~630,000 reads per sample, >88% aligned to the rCRS reference (Table 2).

### 3.2. Nuclear DNA Sensitivity and Size Selection Study

**Figure 3:**
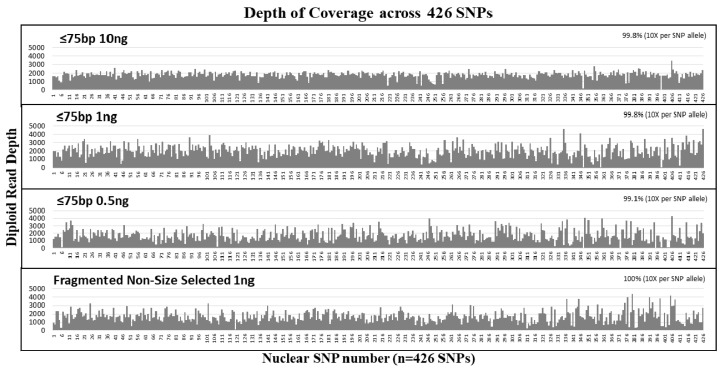
Average diploid read depth per SNP from the size selection study. Mechanically fragmented NA24129 control DNA sample was size selected at ≤75 bp and tested at 10 ng, 1 ng, and 0.5 ng. The diploid reads of each of the 426 SNPs (*y*-axis) was plotted in order of their SNP number (see Table S1 for details of SNPs corresponding to each number (*x*-axis)). All samples exhibited >99% coverage of 426 nuclear SNPs at >10× allelic read depth per SNP.

The sensitivity of our custom SNP probe capture NGS assay to recover nuclear SNPs from short DNA fragments was tested with ≤75 bp size selected samples at input DNA amounts of 10 ng, 1 ng, and 0.5 ng. All size selected samples exhibited an average of ~4.2 million reads per sample. On average, the diploid read depth per SNP was ~1830 per SNP and the average read length per sample was ~79 bases (Table 3). Over 99% of SNPs were captured and sequenced with diploid read depth per SNP allele ≥10× (Figure 3). The depth of coverage was uniformly distributed across all SNPs captured (Figure 3). Comparison with the reference genotype demonstrated no allele drop-ins, and the highest allele drop-out was observed for the ≤75 bp 0.5 ng sample with six SNP alleles that were undetected. At 1 ng input, the number of SNPs without dropouts was nearly equal between the size selected samples and the non-size selected controls. Even after removing the duplicates, the diploid read depths for the 1 ng and 0.5 ng samples were similar at an average of ~200× read depth per SNP. Our results show that over 99% of SNPs were covered for 10 ng and 1 ng and 98.8% of the SNPs were covered for the 0.5 ng sample. Removing PCR duplicates resulted only in 1–2 additional SNP locus dropout (Table 3). These results demonstrate that allelic dropout is more dependent on input DNA copy numbers rather than the size of the DNA targets.

### 3.3. Mock Degradation Study

**Figure 4:**
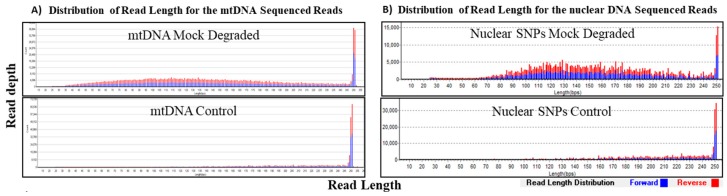
Sequencing results of mock degraded and control samples at 1 ng. Distribution of read length (*x*-axis) at corresponding coverage (*y*-axis) was determined for the mock degraded mtDNA sample and the control sample. Forward reads are in blue and the reverse reads are in red. (**A**) The average read length for the control mtDNA sample was ~200 bp and that of mock degraded mtDNA sample was ~150 bp. (**B**) For the nuclear SNPs control sample, the average read length was ~200 bp and ~150 bp for the mock degraded nuclear SNPs sample.

The recovery of fragmented DNA for both mtDNA and nuclear SNPs using our custom probe capture systems was assessed in the mock degradation study. DNA was mechanically fragmented to an average of 150 bp to mimic degraded DNA and samples exhibited expected size distribution of sequenced reads with average read lengths of 146–147 bp for mtDNA and nuclear SNPs (Table 4, Figure 4). Full coverage of the mtgenome was achieved for both the control and mock degraded mtDNA samples at >100× read depth per base (duplicates not removed), demonstrating capture and sequencing efficiency of DNA fragments independent of fragment size (Table 4).

The total number of reads recovered from the mock degraded and control samples for nuclear SNPs was 2–2.5 million. The average diploid read depth per SNP for the mock degraded sample was 968 and 498 for the control. The two-fold lower read depth for the 1 ng nuclear DNA control resulted in a lower than expected SNP coverage, ~84.5% compared to 96% of the SNPs covered with >10× reads per allele for the mock degraded sample (Table 4). However, even at low diploid read depth, the 96% SNP recovery for the mock degraded sample demonstrates the utility of the system for capturing and sequencing highly fragmented DNA samples.

### 3.4. Mitochondrial DNA and Nuclear SNPs Recovery from Telogen Hairs

**Figure 5:**
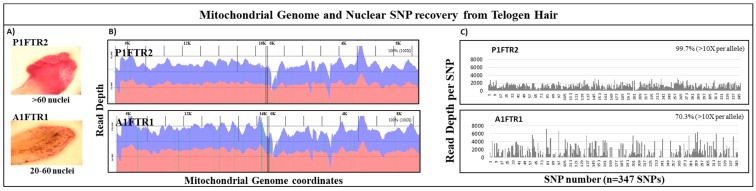
Recovery of mtDNA and nuclear SNPs from telogen hairs. (**A**) Microscopic images of stained telogen hair roots revealed that P1FTR2 exhibited >60 nuclei count while A1FTR1 exhibited 20–60 nuclei count. (**B**) mtgenome read depth plotted against mtgenome coverage coordinates was shown for P1FTR2 and A1FTR1. Blue coverage represents forward reads; red coverage represents reverse reads. Both P1FTR2 and A1FTR1 exhibited 100% coverage of the mtgenome at >100× read depth per base. (**C**) SNP coverage for P1FTR2 and A1FTR1 showed that P1FTR2 exhibited near 100% SNP coverage while A1FTR1 exhibited ~70% SNP coverage. Refer to Table S1 for details of the SNP numbers.

The performance of the mtgenome and nuclear SNP probe capture panels on forensic type samples was assessed using single shotgun DNA libraries constructed from telogen hairs captured in parallel. Analysis of the mtDNA sequencing results for the telogen hair P1FTR2 yielded 100% coverage of the mtgenome at >100× read depth per base (Figure 5B). High specificity of on-target alignment for hair P1FTR2 was achieved; >92% of ~3,900,000 reads were aligned to the rCRS reference (Table 5). Full coverage of the mtgenome was also achieved for the telogen hair A1FTR1 at >100× read depth per base with >96% alignment of ~14 million reads (Figure 5B, Table 5).

Nuclear SNP analysis of the same two hair samples revealed no reads for the Y chromosome SNPs as expected since both hairs were collected from female participants. By excluding the number of Y SNPs (79 SNPs) from the 426 total SNPs, 347 autosomal and X SNPs were analyzed for the two hairs. Sample P1FTR2 with >60 nuclei yielded ~3 million reads with an average diploid read depth of ~1000 reads per SNP while sample A1FTR1 with ~20–60 nuclei yielded ~4 million reads with an average diploid read depth ~1000 reads per SNP (Figure 5A, Table 5). Out of the 347 autosomal and X SNPs, near 100% (346 SNPs) were recovered for P1FTR2 with >60 nuclei and ~70% (244 SNPs) were recovered for A1FTR1 with ~20–60 nuclei at >10× allelic read depth (Figure 5C, Table 5). Using our two custom probe capture panels, one can provide NGS data for both mtDNA and nuclear DNA with high sensitivity and specificity from a single shotgun library for limited forensically challenging samples, such as these two telogen hairs.

### 3.5. Mitochondrial DNA Mixture Study

In the mtDNA mixture study, the performance of the capture assay on recovery of minor contributor mtDNA sequences was assessed with a two-person in vitro mixture. DNA from two contributors of different haplogroups were used to prepare the 80:20 mixture (Caucasian C163 and US Hispanic H104). Analysis of the two-person mixture yielded full coverage of the mtgenome at >100× read depth per base. Sequencing results showed ~86% of the ~1 million reads were aligned to the rCRS reference with an average coverage of ~13,215 coverage per base (data not shown).

**Figure 6:**
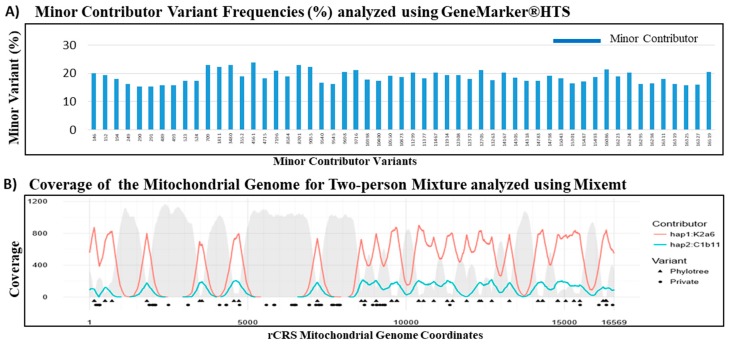
De-convolution of mtDNA mixtures using GeneMarker^®^HTS and Mixemt Software. (**A**) The variant frequency based software GeneMarker^®^HTS successfully separated all minor contributor variants from the 80:20 two-person mixture. (**B**) The phylogenetic based software Mixemt also successfully assigned the sequence reads to major and minor contributors based on the phylogenetic variants (triangles). Red line represents the depth of coverage for reads containing phylogenetic informative variants of haplogroup K2a6, teal line represents the depth of coverage for reads containing phylogenetic informative variants of haplogroup C1b11, and gray areas indicate depth of coverage for the reads that were unassigned. Private mutations (circles) did not affect Mixemt in assigning sequence reads to their respective contributors.

Similar contributor proportion estimation of the 80:20 mixture was observed using both the variant frequency based GeneMarker^®^HTS software and the phylogenetic based Mixemt software. The major and minor mutation reports from the GeneMarker^®^HTS analysis were used for a haplogroup search in EMPOP (EDNAP mtDNA Population Database) [56]. The major contributor with haplogroup K2a6 was estimated to be 79.58% and the minor contributor haplogroup C1b11 at 18.68% using the GeneMarker^®^HTS software (Table 6). All variants pertaining to the minor contributor, including both phylogenetic and private mutations, were accurately assigned by GeneMarker^®^HTS (Figure 6A). Mixemt directly yielded major contributor haplogroup K2a6 at 82.2% and minor contributor haplogroup C1b11 at 17.8%. While Mixemt successfully assigned mtDNA sequence fragments to the contributing major and minor haplogroups based on the phylogenetic variants (marked by triangles in Figure 6B), the software does not consider private mutations (marked by circles in Figure 6B). Overall, both GeneMarker^®^HTS and Mixemt were successful in separating and estimating the major and minor contributors in the observed 80:20 mixture. These results demonstrated the robustness of our probe capture panel to capture minor contributor sequences with ~20% of ~300,000 mtDNA copies. In general, future mixture de-convolution can be most reliably achieved by using both frequency based, when possible, and phylogenetic based software to better characterize and understand private and phylogenetic variants in the human mtgenome.

## 4. Discussion

The clonal and massively parallel features of NGS offer many advantages for the analysis of forensic samples that contain degraded, limited, and/or mixed DNA. One advantage of NGS is that it provides a single platform capable of analyzing mtDNA sequence polymorphism, STRs, and SNPs. However, most strategies for target enrichment rely on the construction of NGS libraries using PCR amplification. Although a relatively simple and efficient method, target enrichment by PCR is not as effective with samples of short DNA fragments as PCR requires the presence of two intact primer binding sites in the template molecule [57].

The hybrid capture method of target enrichment discussed here is capable of analyzing short DNA fragments and therefore, represents a valuable alternative strategy to PCR amplification. Previous studies using PCR based NGS systems have demonstrated limited success in recovering SNPs for samples with template sizes <100 bases [11]. Our experiments using size selected DNA fragments (≤75 bp) and samples sheared to simulate degraded DNA demonstrated that our hybrid probe capture NGS system is capable of recovering and sequencing very short fragments for both mtDNA and a panel of 426 nuclear SNPs (Table 3 and Table 4). In DNA (1 ng input) fragmented to 150 bp (mock degraded), the average of the mtDNA sequence read size distribution was 146 bp, the shortest reads were around 35 bp and 100% coverage (based on >100× read counts per base) of the mtgenome was achieved. Similarly, for nuclear SNPs, the average size of the sequence read distribution was 147 bp, and the shortest reads were around 35 bp, and with 1 ng input, 96% coverage (based on read counts >10× per base) of 426 SNPs was achieved (Table 4). Included in the SNPs with no locus or allele dropout were ≥129 identity informative SNPs (with high heterozygosity); this number of SNPs is much higher than the 50–60 high heterozygosity SNPs that are required for a profile comparable in discriminatory power to the 13 STR [24,58,59,60]. Target enrichment based on a PCR amplicon of, for example, 100 bp would have failed to amplify most of the DNA fragments in the sample. In general, probe capture methods for library preparation combined with the clonal sequencing aspect of NGS are particularly well suited to analysis for mixed samples with short DNA fragments such as the maternal plasma used in non-invasive prenatal testing [26,61].

Although the specificity of hybrid capture is less than that of PCR amplification, our system achieved 100% coverage (>100× read depth per base) for the mtgenome at 10 pg input DNA and an on-target rate of >88% (aligned to the rCRS reference). Off-target reads are then removed using bioinformatics software tools during the alignment process and specificity can be further increased if necessary by adjusting filters and increasing homology cut-offs [62]. In addition to the ability to analyze short DNA fragments, the hybrid capture method of enriching targets from a shotgun library can allow the removal of PCR duplicates, based on the unique start positions of the forward and reverse reads [53]. This feature is critical in evaluating the potential for stochastic variation in sequence read counts and, consequently, in the establishment of thresholds for interpreting sequencing data [33,63]. In Figure 2, the average sequence read coverage per base for the mtDNA genome for an input of 100 pg and of 10 pg was virtually identical (7043 and 7191). However, with PCR duplicates removed, the average number of sequence reads per base was 183 and 22 for the 100 pg and 10 pg samples, respectively. By bioinformatically removing PCR duplicates, we can determine if the sequence data of the initial template DNA molecules were within the stochastic range. Setting thresholds based on the initial copy number can reduce the stochastic effects posed by possible allele dropouts with limited DNA samples during the probe capture enrichment process [15,33].

The NGS analysis of mtDNA sequences has been reported as an effective strategy for the de-convolution of the DNA mixtures characteristic of many forensic specimen [64,65]. The overlapping sequence reads derived from the randomly fragmented DNA in a shotgun library allow for efficient sequence assembly in a single source sample. However, assembling full genome sequences from the short reads obtained by NGS remains challenging for mixtures. In a 90:10 two-person mixture, the high frequency sequence reads can be presumed to be derived from the major contributor and the low frequency reads from the minor contributor. However, if the contributions from the two individuals were more balanced, or if there were more than two contributors to the mixture, then the frequency based assignment of sequence reads to individual contributor becomes problematic. A phylogenetic based software, Mixemt, co-estimates the number of contributors and their contributing proportions, and then assigns sequence reads to each haplogroup based on phylogenetically informative sites. In Figure 6, GeneMarker^®^HTS and Mixemt were both successful in resolving the major and minor contributor haplogroups in the two-person mixture. Furthermore, Vohr et al. demonstrated the ability to resolve a three-person in silico mixture using Mixemt [54]. However, since de-convolution of mixtures using Mixemt is based on phylogenetic variant information, private mutations are not always assigned during this process unless they are found on the same read as phylogenetically informative SNP. While variant frequency based software, such as Genemarker^®^HTS, can resolve mixtures at higher resolution, it is currently impossible to resolve mixtures involving more than two contributors [54]. Thus, the de-convolution of forensic mixtures can be most reliably achieved by using both frequency based and phylogenetic based software to better characterize and understand private and phylogenetic variants.

One of the advantages of the probe capture NGS strategy for analysis of forensically challenging samples is that a single shotgun library construction is needed for both the whole mtgenome and the nuclear SNP panel. This feature is illustrated in the analysis of two telogen hairs (Figure 5). Analysis of the mtDNA sequencing results for both telogen hairs yielded 100% coverage of the mtgenome at >100× read depth with >92% aligned to the rCRS reference (Figure 5). Nuclear SNP analysis (347 autosomal and X SNPs) of the same two hairs yielded an average diploid read depth of ~1000 reads per SNP. Overall, ~99% of the nuclear SNP markers were recovered for sample P1FTR2 and >70% of SNP markers were recovered for sample A1FTR1 at >10× allelic read depth (Table 5). Although SNP loci dropout occurred in both telogen hairs, the partial SNP profile combined with whole mtgenome sequence would increase the discrimination power and likely approach or exceed the discriminatory power of a 13 STR loci profile based on the number of the SNPs recovered (>50–60 high heterozygosity SNPs) [24,58,59,60].

Thus, although the DNA probe capture strategy of target enrichment is less specific than PCR amplification, it provides many advantages for analysis of forensic samples, especially for samples with fragmented DNA. Probe capture enrichment is more effective at recovering and generating sequence reads from short DNA fragments and allows for removal of PCR duplicates to better quantitate the starting copies of the template DNA molecules. As demonstrated here, our custom probe capture panels can provide sensitive analyses of both mtDNA and nuclear DNA from one single shotgun library constructed from a limited forensically challenging sample.

## Figures and Tables

**Table 1:** PCR cycle numbers for varying input DNA amounts.

Total DNA Amount	PCR Cycle Number
≤10 ng–1 ng	13
≤1 ng–500 pg	17
≤500 pg–50 pg	20
≤50 pg–10 pg	24

**Table 2:** Sequencing statistics of mitochondrial DNA sensitivity study samples.

				PCR Duplicates Included		PCR Duplicates Removed	
HL 60 Samples	Input mtDNA Copies	Total Reads	Alignment (%)	Avg. Read Length (bp)	Avg. Coverage Per Base	Avg. Read Length (bp)	Avg. Coverage Per Base
1 ng	200,000	604,052	89.66%	190	6474	181	1226
100 pg	20,000	631,716	89.17%	200	7043	178	183
10 pg	2000	656,556	88.54%	195	7197	157	22

Avg., Average.

**Table 3:** Sequencing statistics for nuclear SNPs size selection study.

			PCR Duplicates Included					PCR Duplicates Removed			
Sample	Input Amount (ng)	Total Reads	Avg. Read Length (bp)	Avg. Diploid Read Depth per SNP	SNP Coverage (*n* = 426 SNPs)	Locus/Allele dropout	Total Reads	Avg. Read Length (bp)	Avg. Diploid Read Depth per SNP	SNP Coverage (*n* = 426 SNPs)	Locus/Allele dropout
**≤75 bp**	10	4,606,916	78	1811 ± 377	425 (99.8%)	1/0	2,476,408	78	517 ± 93	424 (99.5%)	2/0
	1	4,431,052	79	1978 ± 746	425 (99.8%)	1/2	2,638,898	78	217 ± 97	423 (99.3%)	3/1
	0.5	3,679,914	78	1721 ± 820	422 (99.1%)	4/6	2,199,585	78	190 ± 115	421 (98.8%)	5/10
**Control**	1	3,648,297	130*	1690 ± 697	426 (100%)	0/3	2,259,909	127	336 ± 154	425 (99.8%)	1/3

* As a MiSeq Reagent v2 kit (300-cycles, 2 × 150 reads) was used, the maximum sequencing read length was 150 bases.

**Table 4:** Sequencing statistics for mock degraded and control samples at 1 ng.

Mock Degradation Study for mtDNA and nuclear SNPs (Input Amount 1 ng)				
Sample Type	Average Read Length (bp)	Total Reads	Average Depth of Coverage	Coverage (%)
mtDNA Mock Degraded	146	990,269	7148	100 (100×)
mtDNA Control	211	328,256	4130	100 (100×)
Nuclear DNA Mock Degraded	147	2,614,853	968 *	409 (96.0%) **
Nuclear DNA Control	194	1,988,568	498 *	360 (84.5%) **

* Average diploid read depth per SNP, ** percent (%) SNP coverage.

**Table 5:** Sequencing statistics of mtDNA and nuclear SNPs recovery from telogen hairs.

	Nuclei Count	mtDNA Recovery						Nuclear SNPs Recovery				
Sample	Number Nuclei	mtDNA Copies	Avg. Read Length (bp)	Total Reads	Alignment (%)	Avg. Coverage	Coverage ** (%)	nuDNA amt. (ng)	Total Reads	Avg. Read Length (bp)	Avg. Coverage	SNP coverage ***
**P1FTR2**	>60	270,000	185	3,926,604	92.92%	46,624	100	1	3,741,800	204	1035	346 (99.7%) *
**A1FTR1**	20–60	540,000	193	14,015,748	96.14%	184,491	100	1	4,083,881	217	1068	244 (70.3%) *

* As samples were of female origin, the number of SNPs for analysis comprised of 347 autosomal and X SNPs. ** >100× coverage. *** >10× coverage per allele.

**Table 6:** Haplogroup determination and contributor proportions estimation for a contrived 80:20 mixture using GeneMarker^®^HTS and Mixemt Software.

Mixture	Haplogroups		Contributor Proportions (%)	
	GeneMarker^®^HTS	Mixemt	GeneMarker^®^HTS	Mixemt
C163 (Major)	K2a6	K2a6	79.58	82.2
H104 (Minor)	C1b11	C1b11	18.68	17.8

## Supplementary Materials

The following are available online at http://www.mdpi.com/2073-4425/9/1/49/s1.. Table S1: Information Corresponding to SNP Number from Figure 3 and Figure 5.
