# Correction: Shelly Y. Shih; et al.; Applications of Probe Capture Enrichment Next Generation Sequencing for Whole Mitochondrial Genome and 426 Nuclear SNPs for Forensically Challenging Samples. *Genes* 2018, *9*, 49

**DOI:** 10.3390/genes9020090

**Published:** 2018-02-14

**Authors:** Shelly Y. Shih, Nikhil Bose, Anna Beatriz R. Gonçalves, Henry A. Erlich, Cassandra D. Calloway

**Affiliations:** 1 Children’s Hospital Oakland Research Institute, 5700 Martin Luther King Jr. Way, Oakland, CA 94609, USA; sshih@chori.org (S.Y.S.); nbose@ucdavis.edu (N.B.); gonannab@gmail.com (A.B.R.G.); herlich@chori.org (H.A.E.); 2 Forensic Science Graduate Program, University of California, Davis, 1909 Galileo Ct. Ste. B, Davis, CA 95618, USA; 3 Laboratório de Diagnósticos por DNA (LDD), Universidade do Estado do Rio de Janeiro (UERJ), Instituto de Biologia Roberto Alcantara Gomes (IBRAG), Rua São Francisco Xavier, number 524, Pavilhão Haroldo Lisboa da Cunha, Rio de Janeiro 20550-900, RJ, Brazil

The authors wish to make the following change to their paper [1]. Four new references were added: [55] “55. SeqPrep. Available online: https://github.com/jstjohn/SeqPrep (accessed on 13 November 2017)”, [57] “57. Burrow Burrow-Wheeler Aligner. Available online: https://github.com/lh3/bwa (accessed on 13 November 2017)”, [58] “58. SAMTools. Available online: https://github.com/samtools/samtools (accessed on 13 November 2017)” and [59] “59. EMPOP. Available online: https://empop.online/ (accessed on 10 November 2017).”

These references are added in Section 2.4., fourth paragraph, and the text now reads:

“In addition to GeneMarker^®^HTS, the sequenced reads for the mtDNA mixture samples were analyzed using Mixemt, developed by Vohr et al. [54]. Prior to analysis using Mixemt, the raw sequence reads were trimmed for adapters, and the overlapping reads were merged using SeqPrep [55]. Both merged and unmerged sequence reads were then aligned separately to the Reconstructed Sapiens Reference Sequence (RSRS) using the Burrows–Wheeler Aligner (bwa) tool, which also converts the FASTQ files to SAM files [56,57]. SAMtools was then used to collapse the PCR duplicates and convert SAM files to BAM files [58]. The BAM files were used for Mixemt analysis, which assigns each sequence read to a haplogroup based on the probability of the read originating from a contributing haplogroup [50,54].”

Due to this change, the references in the Discussion are renumbered: reference [57] is now [60], reference [58] is now [61], reference [59] is now [62] , reference [60] is now [63] , reference [61] is now [64] , reference [62] is now [65] , reference [63] is now [66] , reference [64] is now [67] and reference [65] is now [68].

We apologize for any inconvenience caused to the readers by this omission. The manuscript will be updated and the original will remain online on the article webpage.

## References

[1] Shih S.Y., Bose N., Gonçalves A.B.R., Erlich H.A., Calloway C.D. (2018). Applications of Probe Capture Enrichment Next Generation Sequencing for Whole Mitochondrial Genome and 426 Nuclear SNPs for Forensically Challenging Samples. Genes.

