# Supplementary material for: Applications of Probe Capture Enrichment Next Generation Sequencing for Whole Mitochondrial Genome and 426 Nuclear SNPs for Forensically Challenging Samples
Source: Genes (Basel). 2018 Jan 22;9(1):49. doi: 10.3390/genes9010049 (PMC5793200; doi:10.3390/genes9010049)
Supplement: Supplementary file 1 [file genes-09-00049-s001.pdf]

Supplement A: Information Corresponding to SNP Number

The information for SNPs corresponding to SNP numbers in x axes of Figure 3 and 5 of the manuscript are listed below. SNP number 1 – 426 correspond to Figure 3 and 1 – 347 correspond to Figure 5.

SNP Number	Chromosome Number	Chromosome Coordinates	RS Number	SNP Category/Type
1	1	3743132	rs4648344	Microhaplotype 1
2	1	3743319	rs6663840	Microhaplotype 1
3	1	4367323	rs1490413	Identity Informative
4	1	14155402	rs7520386	Identity Informative
5	1	15118240	rs1721827	Tetra-allelic
6	1	105717631	rs4847034	Identity Informative
7	1	118165691	rs1630312	Tri-allelic
8	1	151122489	rs7554936	Ancestry Informative
9	1	159174683	rs2814778	Ancestry Informative
10	1	159684665	rs3091244	Tri-allelic
11	1	160786670	rs560681	Identity Informative
12	1	181188091	rs4652604	Tetra-allelic
13	1	208441488	rs1612734	Tetra-allelic
14	1	214634428	rs4528199	Microhaplotype 2
15	1	214634445	rs6604596	Microhaplotype 2
16	1	217236358	rs951416	Tri-allelic
17	1	230820578	rs2296796	Microhaplotype 3
18	1	230820605	rs2296797	Microhaplotype 3
19	1	230820630	rs2296798	Microhaplotype 3
20	1	233448413	rs1294331	Identity Informative
21	1	238439308	rs10495407	Identity Informative
22	1	239881926	rs891700	Identity Informative
23	1	242806797	rs1413212	Identity Informative
24	2	114974	rs876724	Identity Informative
25	2	7968275	rs798443	Ancestry Informative

26	2	10085722	rs1109037	Identity Informative
27	2	17362568	rs1876482	Ancestry Informative
28	2	17901485	rs1834619	Ancestry Informative
29	2	109513601	rs3827760	Ancestry Informative
30	2	109579738	rs260690	Ancestry Informative
31	2	109586313	rs260694	Microhaplotype 4
32	2	109586371	rs11123719	Microhaplotype 4
33	2	109586437	rs11691107	Microhaplotype 4
34	2	124109213	rs993934	Identity Informative
35	2	136707982	rs6754311	Ancestry Informative
36	2	145694454	rs727241	Tri-allelic
37	2	149954097	rs2170607	Microhaplotype 5
38	2	149954253	rs10497052	Microhaplotype 5
39	2	158667217	rs10497191	Ancestry Informative
40	2	177526156	rs2594948	Tetra-allelic
41	2	182413259	rs12997453	Identity Informative
42	2	211456637	rs1047883	Tri-allelic
43	2	239563579	rs907100	Identity Informative
44	3	961782	rs1357617	Identity Informative
45	3	4955595	rs7632479	Tetra-allelic
46	3	32417644	rs4364205	Identity Informative
47	3	46352355	rs4513489	Microhaplotype 6
48	3	46352384	rs6441961	Microhaplotype 6
49	3	46556835	rs6808142	Microhaplotype 7
50	3	46556948	rs17030627	Microhaplotype 7
51	3	59488340	rs9866013	Identity Informative
52	3	113804979	rs1872575	Identity Informative
53	3	113890789	rs3732783	Microhaplotype 8
54	3	113890815	rs6280	Microhaplotype 8
55	3	118272399	rs7610981	Tetra-allelic
56	3	121459589	rs12498138	Ancestry Informative

57	3	133341989	rs35528968	Tri-allelic
58	3	190806108	rs1355366	Identity Informative
59	3	193207380	rs6444724	Identity Informative
60	4	10969059	rs2046361	Identity Informative
61	4	38803255	rs4540055	Tri-allelic
62	4	38815502	rs4833103	Ancestry Informative
63	4	46329655	rs279844	Identity Informative
64	4	67786535	rs13145525	Tetra-allelic
65	4	76425896	rs13134862	Identity Informative
66	4	90673770	rs356167	Tri-allelic
67	4	100239319	rs1229984	Ancestry Informative
68	4	100244319	rs3811801	Ancestry Informative
69	4	100304166	rs2584457	Microhaplotype 9
70	4	100304248	rs12648443	Microhaplotype 9
71	4	100313996	rs2851017	Microhaplotype 10
72	4	100314062	rs2032350	Microhaplotype 10
73	4	100321443	rs4699748	Microhaplotype 11
74	4	100321573	rs2584461	Microhaplotype 11
75	4	100321595	rs1442492	Microhaplotype 11
76	4	105375423	rs7657799	Ancestry Informative
77	4	157489906	rs1554472	Identity Informative
78	4	169663615	rs6811238	Identity Informative
79	4	187538133	rs1280100	Microhaplotype 12
80	4	187538330	rs1280099	Microhaplotype 12
81	4	190318080	rs1979255	Identity Informative
82	5	2879395	rs717302	Identity Informative
83	5	6845017	rs870348	Microhaplotype 13
84	5	6845035	rs870347	Microhaplotype 13
85	5	9619905	rs41461	Microhaplotype 14
86	5	9619936	rs41462	Microhaplotype 14
87	5	17374898	rs159606	Identity Informative

88	5	33951693	rs16891982	Phenotypically Informative
89	5	33958959	rs28777	Phenotypically Informative
90	5	120326716	rs6595279	Tetra-allelic
91	5	136661237	rs13182883	Identity Informative
92	5	155730681	rs6870979	Tetra-allelic
93	5	159487953	rs7704770	Identity Informative
94	5	169735920	rs315791	Identity Informative
95	5	174778678	rs251934	Identity Informative
96	5	178260093	rs6898532	Tetra-allelic
97	5	178690725	rs338882	Identity Informative
98	5	179696997	rs9329104	Tri-allelic
99	6	396321	rs12203592	Phenotypically Informative
100	6	457748	rs4959270	Phenotypically Informative
101	6	1135939	rs1029047	Identity Informative
102	6	12059954	rs13218440	Identity Informative
103	6	16399647	rs4565296	Microhaplotype 15
104	6	16399680	rs4431439	Microhaplotype 15
105	6	16399721	rs179939	Microhaplotype 15
106	6	37554467	rs1738442	Tetra-allelic
107	6	55155704	rs2811231	Identity Informative
108	6	94537255	rs1336071	Identity Informative
109	6	120560694	rs1478829	Identity Informative
110	6	123894978	rs1358856	Identity Informative
111	6	127463376	rs2503107	Identity Informative
112	6	148761456	rs2272998	Identity Informative
113	6	152697706	rs214955	Identity Informative
114	6	165045334	rs727811	Identity Informative
115	7	4310365	rs6955448	Identity Informative
116	7	4457003	rs917118	Identity Informative
117	7	13894276	rs1019029	Identity Informative
118	7	87160618	rs2032582	Tri-allelic

119	7	90518278	rs192655	Ancestry Informative
120	7	96733972	rs17168174	Microhaplotype 16
121	7	96734057	rs10246622	Microhaplotype 16
122	7	137029838	rs321198	Identity Informative
123	7	155990813	rs737681	Identity Informative
124	8	1375610	rs763869	Identity Informative
125	8	11595829	rs1390950	Microhaplotype 17
126	8	11595969	rs2898295	Microhaplotype 17
127	8	16858884	rs4532634	Tri-allelic
128	8	17747876	rs433342	Tri-allelic
129	8	28172586	rs917115	Ancestry Informative
130	8	28411072	rs10092491	Identity Informative
131	8	73352424	rs348146	Tetra-allelic
132	8	110602317	rs6990312	Ancestry Informative
133	8	122124302	rs2196051	Ancestry Informative
134	8	127582924	rs4332095	Tetra-allelic
135	8	136839229	rs4288409	Identity Informative
136	8	144656754	rs4606077	Identity Informative
137	8	145639681	rs1871534	Ancestry Informative
138	9	1823774	rs1015250	Identity Informative
139	9	2032845	rs1112534	Tri-allelic
140	9	12672275	rs1408800	Microhaplotype 18
141	9	12672320	rs1408801	Microhaplotype 18
142	9	12709305	rs683	Phenotypically Informative
143	9	14747133	rs2270529	Identity Informative
144	9	27985938	rs7041158	Identity Informative
145	9	110085992	rs3818367	Tri-allelic
146	9	126881448	rs1463729	Identity Informative
147	9	127267689	rs3814134	Ancestry Informative
148	9	128968063	rs1360288	Identity Informative
149	9	137417115	rs3118582	Microhaplotype 19

150	9	137417308	rs10776839	Identity Informative/Microhaplotype 19
151	10	2406631	rs826472	Identity Informative
152	10	3374178	rs735155	Identity Informative
153	10	15600703	rs9333212	Tri-allelic
154	10	17193346	rs3780962	Identity Informative
155	10	54530788	rs17287498	Tri-allelic
156	10	88425555	rs2803554	Tri-allelic
157	10	94921065	rs4918664	Ancestry Informative
158	10	97172595	rs1410059	Identity Informative
159	10	118506899	rs740598	Identity Informative
160	10	132698419	rs964681	Identity Informative
161	11	2168375	rs11042874	Tri-allelic
162	11	5098714	rs10768550	Identity Informative
163	11	5099393	rs10500617	Identity Informative
164	11	5109946	rs10500616	Microhaplotype 20
165	11	5110068	rs2499936	Microhaplotype 20
166	11	5709028	rs1498553	Identity Informative
167	11	11096221	rs901398	Identity Informative
168	11	32424389	rs5030240	Tri-allelic
169	11	61597212	rs174570	Ancestry Informative
170	11	88911696	rs1042602	Phenotypically Informative
171	11	89011046	rs1393350	Phenotypically Informative
172	11	105912984	rs6591147	Identity Informative
173	11	113111501	rs2303377	Microhaplotype 21
174	11	113111665	rs2303378	Microhaplotype 21
175	11	113211329	rs2288159	Microhaplotype 22
176	11	113211370	rs10891537	Microhaplotype 22
177	11	113264470	rs4938013	Microhaplotype 23
178	11	113264511	rs11214596	Microhaplotype 23
179	11	113283459	rs6277	Microhaplotype 24
180	11	113283477	rs6275	Microhaplotype 24

181	11	113289182	rs1079727	Microhaplotype 25
182	11	113289298	rs2002453	Microhaplotype 25
183	11	113296286	rs1079597	Ancestry Informative
184	11	115207176	rs10488710	Identity Informative
185	11	122195989	rs590162	Identity Informative
186	11	134667546	rs2076848	Identity Informative
187	12	888320	rs2107612	Identity Informative
188	12	6909442	rs2255301	Identity Informative
189	12	6945914	rs2269355	Identity Informative
190	12	40863052	rs2920816	Identity Informative
191	12	80014907	rs2307223	Tri-allelic
192	12	89328335	rs12821256	Phenotypically Informative
193	12	103260634	rs2133298	Microhaplotype 26
194	12	103260819	rs3817446	Microhaplotype 26
195	12	106328254	rs2111980	Identity Informative
196	12	112211833	rs2238151	Ancestry Informative
197	12	118889488	rs1503767	Microhaplotype 27
198	12	118889559	rs11068953	Microhaplotype 27
199	12	130761696	rs10773760	Identity Informative
200	13	20901724	rs1335873	Identity Informative
201	13	22374700	rs1886510	Identity Informative
202	13	24530134	rs2765117	Tetra-allelic
203	13	41715282	rs1572018	Ancestry Informative
204	13	42579985	rs2166624	Ancestry Informative
205	13	49070512	rs7326934	Ancestry Informative
206	13	50887560	rs806301	Microhaplotype 28
207	13	50887725	rs2066700	Microhaplotype 28
208	13	84456735	rs9546538	Identity Informative
209	13	91171678	rs1929060	Tetra-allelic
210	13	100038233	rs1058083	Identity Informative
211	13	106938411	rs354439	Identity Informative

212	13	111827167	rs9522149	Ancestry Informative
213	14	21367444	rs1008686	Tri-allelic
214	14	25850832	rs1454361	Identity Informative
215	14	46924415	rs28674745	Tetra-allelic
216	14	53216723	rs722290	Identity Informative
217	14	74250557	rs12717560	Microhaplotype 29
218	14	74250715	rs12878166	Microhaplotype 29
219	14	92773663	rs12896399	Phenotypically Informative
220	14	92801203	rs2402130	Phenotypically Informative
221	14	99375321	rs200354	Ancestry Informative
222	14	104769149	rs4530059	Identity Informative
223	15	24571796	rs2016276	Identity Informative
224	15	28187772	rs1545397	Phenotypically Informative
225	15	28197037	rs1800414	Ancestry Informative
226	15	28230318	rs1800407	Phenotypically Informative
227	15	28365618	rs12913832	Ancestry Informative/Phenotypically Informative
228	15	39313402	rs1821380	Identity Informative
229	15	39842174	rs8024606	Tetra-allelic
230	15	45152371	rs735480	Ancestry Informative
231	15	48426484	rs1426654	Ancestry Informative
232	15	53616909	rs8037429	Identity Informative
233	15	55210705	rs1528460	Identity Informative
234	15	101934168	rs58329356	Tetra-allelic
235	16	5606197	rs729172	Identity Informative
236	16	5868700	rs2342747	Identity Informative
237	16	7520254	rs7205345	Identity Informative
238	16	55540756	rs11865501	Tri-allelic
239	16	56926828	rs2278489	Tri-allelic
240	16	78017051	rs430046	Identity Informative
241	16	80106361	rs1382387	Identity Informative
242	16	88241056	rs67406039	Tetra-allelic

243	16	89730827	rs459920	Ancestry Informative
244	16	89985844	rs1805005	Phenotypically Informative
245	16	89985918	rs1805006	Phenotypically Informative
246	16	89985940	rs2228479	Phenotypically Informative
247	16	89986091	rs11547464	Phenotypically Informative
248	16	89986117	rs1805007	Phenotypically Informative
249	16	89986130	rs1110400	Phenotypically Informative
250	16	89986144	rs1805008	Phenotypically Informative
251	16	89986154	rs885479	Phenotypically Informative
252	17	2919393	rs9905977	Identity Informative
253	17	5706623	rs740910	Identity Informative
254	17	6811529	rs4796362	Identity Informative
255	17	7554536	rs1050528	Tri-allelic
256	17	40658533	rs4411548	Ancestry Informative
257	17	41056245	rs2593595	Ancestry Informative
258	17	41286822	rs2175957	Identity Informative
259	17	41341984	rs8070085	Identity Informative
260	17	41691526	rs1004357	Identity Informative
261	17	43472321	rs1059504/rs114063948	Microhaplotype 30
262	17	43472507	rs8327	Microhaplotype 30
263	17	45695832	rs3760370	Microhaplotype 31
264	17	45695914	rs3760371	Microhaplotype 31
265	17	46510697	rs1027895	Identity Informative
266	17	47287067	rs2233362	Microhaplotype 32
267	17	47287109	rs634370	Microhaplotype 32
268	17	48112927	rs6504633	Tetra-allelic
269	17	62987151	rs11652805	Ancestry Informative
270	17	68849814	rs7218712	Tetra-allelic
271	17	80461935	rs8078417	Identity Informative
272	17	80526139	rs2291395	Identity Informative
273	17	80531643	rs4789798	Identity Informative

274	17	80715702	rs689512	Identity Informative
275	17	80739859	rs3744163	Identity Informative
276	17	80765788	rs2292972	Identity Informative
277	18	1127986	rs1493232	Identity Informative
278	18	9749879	rs9951171	Identity Informative
279	18	22739001	rs7229946	Identity Informative
280	18	29311034	rs985492	Identity Informative
281	18	35277622	rs2042762	Ancestry Informative
282	18	47371014	rs521861	Identity Informative
283	18	55225777	rs1736442	Identity Informative
284	18	67867663	rs4891825	Ancestry Informative
285	18	75432386	rs1024116	Identity Informative
286	19	4077096	rs7251928	Ancestry Informative
287	19	4852137	rs1055919	Microhaplotype 33
288	19	4852337	rs2271057	Microhaplotype 33
289	19	39559807	rs576261	Identity Informative
290	19	44163746	rs385780	Tri-allelic
291	20	4447483	rs1031825	Identity Informative
292	20	12632017	rs12625560	Tetra-allelic
293	20	15124933	rs445251	Identity Informative
294	20	16241416	rs12480506	Identity Informative
295	20	23017082	rs2567608	Identity Informative
296	20	29550804	rs12481195	Tetra-allelic
297	20	32785212	rs6119471	Phenotypically Informative
298	20	33218090	rs2378249	Phenotypically Informative
299	20	33761837	rs2069945	Tri-allelic
300	20	39487110	rs1005533	Identity Informative
301	20	51296162	rs1523537	Identity Informative
302	20	59703918	rs10854214	Microhaplotype 34
303	20	59704014	rs10854215	Microhaplotype 34
304	21	16685598	rs722098	Identity Informative

305	21	28023370	rs464663	Identity Informative
306	21	28608163	rs2830795	Identity Informative
307	21	29679687	rs2831700	Identity Informative
308	21	33582722	rs2833736	Identity Informative
309	21	34392683	rs35474228	Tetra-allelic
310	21	36590104	rs471010	Tri-allelic
311	21	42415929	rs914165	Identity Informative
312	21	43606997	rs221956	Identity Informative
313	22	19920359	rs9606186	Identity Informative
314	22	19920646	rs5746846	Identity Informative
315	22	19951207	rs4818	Microhaplotype 35
316	22	19951271	rs4680	Microhaplotype 35
317	22	23802171	rs2073383	Identity Informative
318	22	27816784	rs733164	Identity Informative
319	22	33559508	rs987640	Identity Informative
320	22	41697338	rs2024566	Ancestry Informative
321	22	46656247	rs34741930	Tri-allelic
322	22	47836412	rs2040411	Identity Informative
323	22	48362290	rs1028528	Identity Informative
324	X	4066743	rs2056688	X Chromosomal
325	X	5616964	rs2128519	X Chromosomal
326	X	11882557	rs1534285	X Chromosomal
327	X	18151389	rs763056	X Chromosomal
328	X	25779585	rs1373592	X Chromosomal
329	X	34091679	rs993010	X Chromosomal
330	X	35632777	rs1557054	X Chromosomal
331	X	40871567	rs1243792	X Chromosomal
332	X	42848162	rs925178	X Chromosomal
333	X	47686005	rs1207480	X Chromosomal
334	X	67200648	rs1936313	X Chromosomal
335	X	94465580	rs1372687	X Chromosomal

336	X	95114611	rs1857602	X Chromosomal
337	X	104268001	rs985425	X Chromosomal
338	X	106598707	rs933315	X Chromosomal
339	X	116521416	rs2190288	X Chromosomal
340	X	119826608	rs1991961	X Chromosomal
341	X	120864696	rs1931662	X Chromosomal
342	X	126325138	rs149910	X Chromosomal
343	X	128259481	rs1573704	X Chromosomal
344	X	139920048	rs1340718	X Chromosomal
345	X	144091597	rs1930674	X Chromosomal
346	X	145069663	rs1339597	X Chromosomal
347	X	151150133	rs1981452	X Chromosomal
348	Y	2663943	rs9786608	Y Chromosomal
349	Y	2734854	rs35284970	Y Chromosomal
350	Y	2828425	rs2267802	Y Chromosomal
351	Y	3545167	rs2350606	Y Chromosomal
352	Y	6000464	rs7067483	Y Chromosomal
353	Y	6965215	rs16981311	Y Chromosomal
354	Y	7218079	rs4988808	Y Chromosomal
355	Y	7395806	rs9786877	Y Chromosomal
356	Y	7568568	rs16981290	Y Chromosomal
357	Y	7570822	rs1005041	Y Chromosomal
358	Y	7963031	rs895530	Y Chromosomal
359	Y	8050994	rs9786915	Y Chromosomal
360	Y	8602415	rs16981340	Y Chromosomal
361	Y	8679843	rs3853054	Y Chromosomal
362	Y	8685083	rs4141564	Y Chromosomal
363	Y	9866740	rs16980711	Y Chromosomal
364	Y	14197867	rs4141886	Y Chromosomal
365	Y	14199284	rs16980478	Y Chromosomal
366	Y	14432928	rs2740980	Y Chromosomal

367	Y	14497207	rs60115999	Y Chromosomal
368	Y	14664631	rs9786634	Y Chromosomal
369	Y	14790163	rs3913	Y Chromosomal
370	Y	14804077	rs9786191	Y Chromosomal
371	Y	14847792	rs2032597	Y Chromosomal
372	Y	14851554	rs2032599	Y Chromosomal
373	Y	14898163	rs20320	Y Chromosomal
374	Y	14904859	rs2032607	Y Chromosomal
375	Y	14954280	rs2032602	Y Chromosomal
376	Y	15014506	rs2032676	Y Chromosomal
377	Y	15018582	rs8179021	Y Chromosomal
378	Y	15020578	rs9341290	Y Chromosomal
379	Y	15021522	rs9786025	Y Chromosomal
380	Y	15023364	rs9341301	Y Chromosomal
381	Y	15027529	rs2032636	Y Chromosomal
382	Y	15415115	rs16980601	Y Chromosomal
383	Y	15437564	rs2032666	Y Chromosomal
384	Y	15469724	rs9341278	Y Chromosomal
385	Y	15472863	rs9786043	Y Chromosomal
386	Y	15481372	rs9341277	Y Chromosomal
387	Y	15508706	rs2032678	Y Chromosomal
388	Y	15581983	rs2032658	Y Chromosomal
389	Y	15754313	rs917759	Y Chromosomal
390	Y	15809326	rs16980360	Y Chromosomal
391	Y	15935524	rs7067279	Y Chromosomal
392	Y	16251357	rs16980586	Y Chromosomal
393	Y	16338537	rs9786301	Y Chromosomal
394	Y	16846439	rs9786893	Y Chromosomal
395	Y	17285993	rs17307398	Y Chromosomal
396	Y	17420017	rs9786126	Y Chromosomal
397	Y	17470112	rs9786781	Y Chromosomal

398	Y	17508337	rs17222419	Y Chromosomal
399	Y	17716251	rs1118473	Y Chromosomal
400	Y	18028661	rs17307656	Y Chromosomal
401	Y	18656508	rs9785702	Y Chromosomal
402	Y	18964263	rs17315758	Y Chromosomal
403	Y	19179335	rs17315835	Y Chromosomal
404	Y	19267344	rs17249974	Y Chromosomal
405	Y	19500107	rs9786357	Y Chromosomal
406	Y	20337461	rs2032623	Y Chromosomal
407	Y	20366926	rs2032651	Y Chromosomal
408	Y	21083420	rs16980577	Y Chromosomal
409	Y	21618583	rs16980574	Y Chromosomal
410	Y	21722998	rs3901	Y Chromosomal
411	Y	21733454	rs3911	Y Chromosomal
412	Y	21751449	rs34289137	Y Chromosomal
413	Y	21778998	rs9306841	Y Chromosomal
414	Y	21843090	rs891407	Y Chromosomal
415	Y	21893881	rs2032672	Y Chromosomal
416	Y	21894058	rs2032673	Y Chromosomal
417	Y	21917313	rs2032652	Y Chromosomal
418	Y	22072340	rs4116821	Y Chromosomal
419	Y	22094491	rs3865828	Y Chromosomal
420	Y	22738775	rs9341308	Y Chromosomal
421	Y	22745051	rs9341318	Y Chromosomal
422	Y	22749853	rs13447352	Y Chromosomal
423	Y	22750583	rs1558843	Y Chromosomal
424	Y	22918577	rs16980548	Y Chromosomal
425	Y	23035132	rs9786261	Y Chromosomal
426	Y	23443971	rs17842518	Y Chromosomal
